# Stereo Visual Servoing Control of a Soft Endoscope for Upper Gastrointestinal Endoscopic Submucosal Dissection †

**DOI:** 10.3390/mi15020276

**Published:** 2024-02-15

**Authors:** Jian Chen, Shuai Wang, Qingxiang Zhao, Wei Huang, Mingcong Chen, Jian Hu, Yihe Wang, Hongbin Liu

**Affiliations:** 1 School of Artificial Intelligence, University of Chinese Academy of Sciences, Beijing 100049, China; chenjian2020@ia.ac.cn; 2 Institute of Automation, Chinese Academy of Sciences, Beijing 100190, China; hujian@ia.ac.cn; 3 Centre of AI and Robotics, Hong Kong Institute of Science and Innovation, Chinese Academy of Sciences, Hong Kong; shuai.wang@cair-cas.org.hk (S.W.); qingxiang.zhao@cair-cas.org.hk (Q.Z.); wei.huang@cair-cas.org.hk (W.H.); yihe.wang@cair-cas.org.hk (Y.W.); 4 Department of Biomedical Engineering, City University of Hong Kong, Hong Kong; mingcong.chen@my.cityu.edu.hk; 5 School of Biomedical Engineering and Imaging Sciences, King’s College London, London SE1 7EU, UK

**Keywords:** surgical robot, stereo visual servoing control, endoscopic submucosal dissection, soft robot

## Abstract

Quickly and accurately completing endoscopic submucosal dissection (ESD) operations within narrow lumens is currently challenging because of the environment’s high flexibility, invisible collision, and natural tissue motion. This paper proposes a novel stereo visual servoing control for a dual-segment robotic endoscope (DSRE) for ESD surgery. Departing from conventional monocular-based methods, our DSRE leverages stereoscopic imaging to rapidly extract precise depth data, enabling quicker controller convergence and enhanced surgical accuracy. The system’s dual-segment configuration enables agile maneuverability around lesions, while its compliant structure ensures adaptability within the surgical environment. The implemented stereo visual servo controller uses image features for real-time feedback and dynamically updates gain coefficients, facilitating rapid convergence to the target. In visual servoing experiments, the controller demonstrated strong performance across various tasks. Even when subjected to unknown external forces, the controller maintained robust performance in target tracking. The feasibility and effectiveness of the DSRE were further verified through ex vivo experiments. We posit that this novel system holds significant potential for clinical application in ESD surgeries.

## 1. Introduction

**Figure 1:**
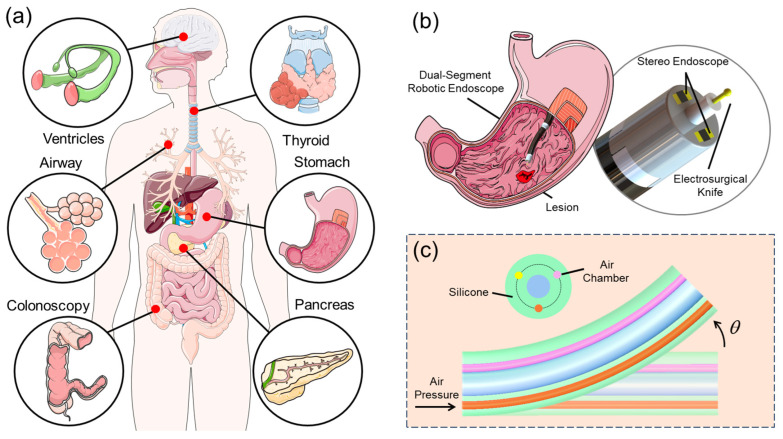
(**a**) Flexible endoscopes provide effective minimally invasive treatment through different naturally narrow orifices in the human body. (**b**) DSRE promotes dexterity and flexibility for ESD, and stereo vision contributes to evaluation before and after surgery. (**c**) Schematic diagram of the DSRE silicone-body soft segment, which would bend when the air pressure in the chamber increases.

Gastrointestinal (GI) cancer is a prevalent and highly malignant tumor in clinical practice [1]. Stomach cancer, in particular, ranks third in terms of mortality due to its low survival rate, with 782,685 deaths reported in 2018 [2]. Improving the survival rate of GI cancer relies heavily on early-stage treatment [3,4]. Owing to the high compliance, control precision, and cost-effectiveness, flexible endoscopes are increasingly being utilized in a variety of minimally invasive surgical procedures (see Figure 1a). Since the first application in 1988 [5], endoscopic submucosal dissection (ESD) has become the gold standard for the early treatment of GI cancer [6]. ESD enables the en bloc resection of submucosal lesions, resulting in a lower recurrence rate [7]. Currently, most ESD procedures are dependent on systems that utilize a single flexible arm outfitted with a monocular endoscope and various electrosurgical knives designed for specific surgical tasks [8]. However, due to the limited degrees of freedom (DoFs) of existing ESD endoscopes and the high skills required, improper operations during the procedure may lead to some complications such as tissue perforation and bleeding [9]. Additionally, the narrow visual field offered by traditional endoscopes hinders the surgeon’s ability to perform precise, multi-angle excisions, emphasizing the necessity for an ESD system with improved visual capabilities and instrument control [10].

To simplify the procedure and improve maneuverability, researchers have proposed different robot arm designs by adapting conventional surgical tools, enabling novices to perform the surgery easily. Chiu et al. [11] compared the feasibility and proficiency of ESD using an insulation-tipped (IT) diathermic knife, demonstrating that the robotic ESD device MASTER is more effective in terms of time cost and exhibits a good grasping and cutting efficiency [12]. Other similar ESD devices include ESD+AWC [13], K-FLEX [14], and TURBT [15]. However, the monocular vision system on traditional ESD endoscopes limits depth perception and lacks the stereoscopic perception of the lesion, which may increase the surgical operation time [16] and compromise the accuracy of resection [17,18]. The application of stereoscopic vision in various miniaturized surgical instruments, such as the da Vinci surgical system [19] and Versius surgical robot [20], enhances surgeons’ hand–eye coordination and depth judgment capabilities in minimally invasive surgeries. Figure 1b illustrates a dual-segment robotic endoscope (DSRE) [21] with a stereo vision system, which is used to reach the stomach lesion through the esophagus and perform the excision. As shown in Figure 1c, the DSRE consists of two air-driven soft segments that can be actively bent by tuning the air pressure.

Challenges remain in controlling the end effector to achieve a desired pose in a flexible continuum robot arm, especially given the hyper-redundancy and infinite DoFs [22]. Contact with the target also introduces disturbances at the tip and the circumferential body. Therefore, accurate modeling of the relationship between the actuation inputs, tip pose, and compensation for disturbances is essential. Although modeling in continuum robotics is a well-explored topic [23], the design of an eye-in-hand system like a flexible endoscope is not common. The stereo vision, embedded at the robot tip, contributes to image-based visual servoing (IBVS), enhancing the system’s compatibility with the eye-in-hand setup [24]. Table 1 summarizes the related research on multi-segment soft robot control, highlighting the superiority of visual servoing for robot operation in unstructured environments. The classical IBVS has been widely used to solve the tracking [25], shape control [26], and depth estimation [27] problems of flexible endoscopes. During endoscopic operations, external interference, such as inserting internal instruments, may lead to potential issues with the proportional controller. These issues could manifest as slow convergence [28] and decreased tracking performance [25]. To overcome these uncertainties, control algorithms such as sliding mode control [29], model predictive control [30], and multi-sensor fusion [31] are deployed in the IBVS framework to improve system robustness. The camera not only provides a view of the lesion but also senses the actual motion of the robot arm, serving as a proprioception mechanism for disturbance compensation. In [31], the depth of the endoscopic image is extracted for camera position adjustment in laparoscopic surgeries. In this study, stereo vision is employed to capture a wide view of the lesion and estimate the depth between the robot tip and the desired target, providing sensing information for disturbance compensation.

This study presents a dual-segment robotic endoscope with a control algorithm based on binocular visual servoing, and preliminarily discusses the application value of stereo vision in ESD surgery. To address the extant clinical challenges, the design requirements of the DSRE are introduced in detail in Section 2, and the fabrication procedure of the robot and the design of the pneumatic control unit are explained, respectively. Section 3 explains the modeling of the relationship between the actuation inputs of the robot and the tip pose, as well as the approach to sensing an object in 3D form. Additionally, Section 3 introduces an adaptive stereo visual servoing (ASVS)-based control scheme. The experiment results in Section 4 demonstrate the effectiveness of the robot and the proposed methods through controller tests and an ex vivo ESD trial. Finally, Section 5 concludes this work. Compared with a previously published conference paper [21], the main improvements in this expanded study include a smaller and more flexible robot for ESD with segments calibration, the ASVS controller with adaptive gain and PD control, and a more comprehensive experimental validation. 

## 2. System Design

### 2.1. Robot Design

The DSRE is designed to function the same as the distal tip [37] on a traditional endoscope; thus, it was designed with the following three goals in mind: (i) to allow for multiple orientations along a resection path, as a single segment proves inadequate; (ii) to fit within the adult esophagus, which measures 25 cm in length and 2 cm in diameter [38]; and (iii) to facilitate a wide viewing angle during surgery and the efficient operation of a T-type electrosurgical cutting knife.

**Figure 2:**
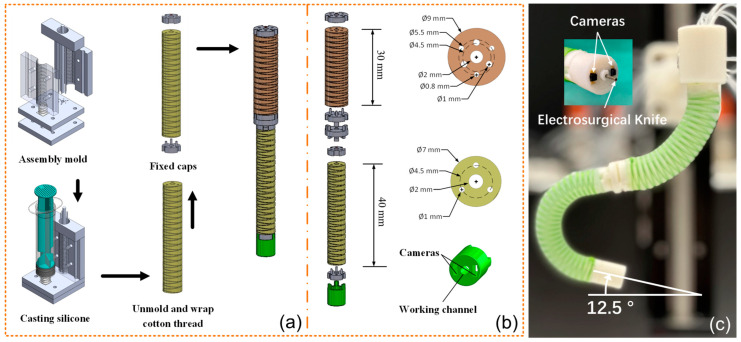
(**a**) Fabrication process of the DSRE. (**b**) Detailed dimension. (**c**) DSRE with two cameras and an electrosurgical knife.

To this end, both the proximal and distal segment of the DSRE were designed to have three actuators longitudinally aligned with the main body and have a 2 mm through working channel for the electrosurgical knife or biopsy grasper, as shown in Figure 2b. When fabricated with appropriate pretension, this construction allows for approximately 170° and 88° of curvature when the proximal and distal are fully actuated, respectively. More detailed dimensions of the DSRE are shown in Figure 2b. As shown in Table 2, compared with the previous design [21] and conventional endoscopes, the DSRE’s robotic actuator can provide more precise control, while its smaller size and increased bending capabilities expand the operational area within the gastrointestinal tract. Additionally, the stereo vision provides supplementary depth information.

Two commercial cameras (OV6946, OmniVision, Santa Clara, CA, USA) with a distribution distance of 4.4 mm were attached at the tip, providing a wide view angle during surgery, as shown in Figure 2c. Similarly, a T-type electrosurgical cutting knife was fixed at the tip, acting as the end effector of the robot arm. The resection power and mode could be set on an electrosurgical generator (DGD-300B-2, Beilin, Beijing, China).

### 2.2. Robot Fabrication

As shown in Figure 2a, by casting the prototypes on 3D-printed molds, the soft segments were constructed of two low-stiffness silicones, Ecoflex 0050 and Dragon Skin 10 AF (Smooth-On, Macungie, PA, USA) mixed in a 1:1 ratio. To restrict elongation and limit radial expansion when pressurized, a nylon thread was attached at the end of each segment and another thread was coiled spirally. The DSRE of two serially connected segments was then affixed to a linear stage to facilitate insertion into the stomach.

### 2.3. Pneumatic Control Design

Pneumatic actuation is used in the DSRE system for its high precision control, quick response, safety for medical use, and the adaptability of in-body applications. Each air chamber of the robotic arm was linked to a pneumatic regulator (ITV0030, SMC, Tokyo, Japan) by a 60 cm silicone tube (0.3 mm inner, 0.8 mm outer diameter), and command individual pressures continuously to bend the DSRE with a direction angle and a bending angle. These valves were chosen for the following reasons: fast response times (0.1 s with no load), high stability (within 0.5% full span repeatability error), and accurate response (within 1% full span linearity error).

### 2.4. Robot Calibration

The calibration of soft robots is an essential process that helps determine the mechanical behavior under different loads and conditions, enabling better control and manipulation. To verify the mechanical performance and chamber consistency of the proposed dual-segment soft robot, we initially examined its bending behavior corresponding to each independent air chamber.

**Figure 3:**
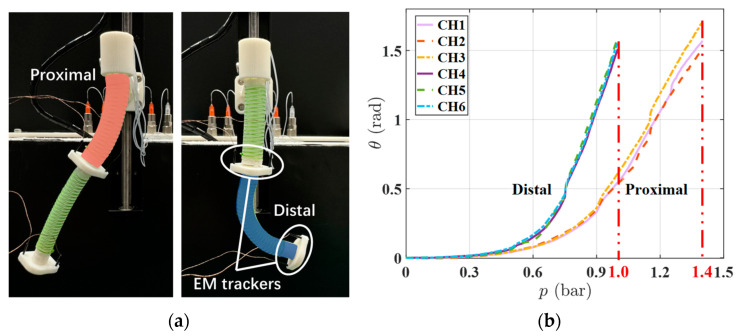
(**a**) Configuration of the EM trackers on proximal and distal segments. (**b**) Bending angles of proximal and distal segments corresponding to the air pressure, the red lines correspond to the maximum air pressure exerted on each segment.

Figure 3a illustrates the configurations of the electromagnetic (EM) trackers located at the proximal and distal segments of the DSRE. Notably, to accurately obtain the position and orientation of each segment center, two trackers were symmetrically affixed to a 3D-printed part mounted at the proximal/distal segment’s end. We subsequently processed the average of the sensed data to calculate the positions and Euler angles. During testing, the pneumatic regulator was controlled to gradually increase the air pressure with a constant increment of 5000 Pa, while simultaneously recording the bending angles. As shown in Figure 3b, the curves of the bending angles corresponding to each segment’s three chambers were roughly equivalent. When reaching the maximum air pressure (0.14 MPa for the proximal segment and 0.1 MPa for the distal segment), the standard deviations of the bending angles across the different chambers were remarkably low, which were only 0.1012 rad and 0.0428 rad, respectively. These results illustrate that the soft robot has a consistent and stable bending performance in all directions.

## 3. Methodology

Through geometric analysis and an optimization algorithm, the modeling was first established, and the two endoscopes placed in an embedded sensing system to compensate for the external load effect.

### 3.1. Kinematics

#### 3.1.1. Shape to Tip Pose

**Figure 4:**
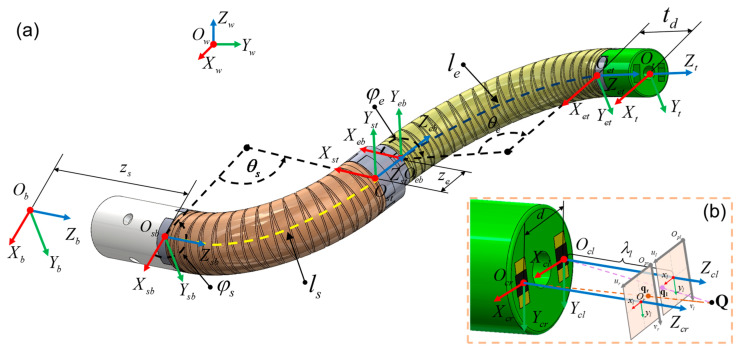
(**a**) Illustration of coordinate frames. (**b**) The binocular stereo vision on the tip.

For soft segments with a large slenderness ratio, when the eccentrically fixed chamber is subjected to changing air pressure, the robot will bend and deform. Referring to the PCC model proposed in [42], it is assumed that the bending shape of each soft robot on the DSRE is a circular arc. As shown in Figure 4a, the DSRE can be geometrically parameterized by $\Phi ={\left(\begin{array}{ccccc} z_{s} & \theta_{s} & \varphi_{s} & \theta_{e} & \varphi_{e} \end{array}\right)}^{T}$ in the configuration space, where $z_{s}$ is the overall insert distance provided by a linear stage, $\theta_{s}$ and $\theta_{e}$ are the bending angles, $\varphi_{s}$ and $\varphi_{e}$ are the direction angles between the bending plane and the *oxz* plane, $l_{s}$ and $l_{e}$ are the length of the proximal and distal segment, and $z_{e}$ and $t_{d}$ are the length of the middle cap and tip cap. 

Taking the proximal segment as an example, the coordinate transformation sequence from the $O_{sb}X_{sb}Y_{sb}Z_{sb}$ to $O_{st}X_{st}Y_{st}Z_{st}$ coordinate system is as follows: first rotate the $\varphi_{s}$ angle around the *z*-axis, then translate $l_{s}/\theta_{s}$ along the *x*-axis, then rotate the $\theta_{s}$ angle around the *y*-axis, and, finally, translate $l_{s}/\theta_{s}$ along the negative direction of the *x*-axis. The corresponding homogeneous transformation matrix is calculated as follows:

(1) Tstsb=Rz(φs)τx(lsθs)Ry(θs)τx(−lsθs)

where $\tau_{*},\;R_{*}\in {\mathbb{R}}^{4\times 4}$, respectively, denote the translation and rotation about the {x,y,z} axis. 

Considering the proximal segment and the displacements $z_{s}$, $z_{e}$, and $t_{d}$, the overall transformation matrix Ttb∈ℝ4×4 from the base frame $O_{b}X_{b}Y_{b}Z_{b}$ to the robot tip frame $O_{t}X_{t}Y_{t}Z_{t}$ of the DSRE system is as follows:

(2) Ttb=τz(zs)Rz(φs)τx(lsθs)Ry(θs)τx(−lsθs)Rz(π3+φe)τz(ze)Rz(φe)τx(leθe)Ry(θe)τx(−leθe)τz(td)

where the shift angle between the two segments is 60° 

The Jacobian matrix $J_{r}\in {\mathbb{R}}^{6\times 5}$ is used to analytically establish the approximate relationship between the robot tip velocity $v_{t}\in {\mathbb{R}}^{6}$ and joint velocity $\dot{\Phi }$:

(3) $$v_{t}=J_{r}\dot{\Phi }$$

where $J_{r}$ can be derived through forward kinematics Ttb. A more detailed calculation of the forward kinematics and the Jacobian matrix can be found in Appendix A.

To reach a given target position of the end of the robot, we need to inversely solve the appropriate joint configuration. 

#### 3.1.2. Air Pressure to Shape

After finding the shape parameters, the robot system should set proper air pressures for each segment. The joint angles $\theta_{s}$, $\theta_{e}$, $\varphi_{s}$, and $\varphi_{e}$ can be calculated from the actuator space variables $q={\left(\begin{array}{ccccccc} z_{s} & p_{s,1} & p_{s,2} & p_{s,3} & p_{e,1} & p_{e,2} & p_{e,3} \end{array}\right)}^{T}$:

(4) $$\begin{array}{l} \theta_{i}=\frac{2\sqrt{p_{i,1}^{2}+p_{i,2}^{2}+p_{i,3}^{2}-p_{i1}p_{i2}-p_{i2}p_{i3}-p_{i1}p_{i3}}}{3\rho_{i}}\\ \varphi_{i}=\tan^{-1}\left(\frac{p_{i1}+p_{i3}-2p_{i2}}{\sqrt{3}\left(p_{i3}-p_{i1}\right)}\right) \end{array}$$

where i∈{e,s}, and the subscripts *e* and *s* are used to represent the proximal and distal segment, respectively, $\rho_{i}$ is the distance between the center of the air chamber and the center of the segment, $p_{i,m},\;m\in \left\{1,2,3\right\}$ are the air pressure of the chambers in each segment.

### 3.2. Depth Estimation

The two cameras mounted at the robot tip could not only view the lesion with a wide angle but also could be employed to calculate the depth of a target (vertical distance from camera to the target). In this study, we employed the semi-global matching (SGM) algorithm [43], renowned for its efficacy in disparity estimation. From the disparity and stereo camera intrinsic information, the targets’ 3D position relative to camera frames can be estimated. The SGM algorithm encompasses a pixelwise matching technique based on mutual information (MI) [44] and a semi-global 2D constraint, composed of multiple 1D constraints. It is detailed as follows.

#### 3.2.1. Mutual Information-Based Cost function

Initially, SGM computes the similarity between pixels in two rectified stereo images using a mutual information (MI)-based cost function, which is less sensitive to photometric changes. Thus, the MI value can be used to evaluate the suitability of pixels. The higher the value, the better the match, and vice versa. For the left and right images $I_{l}$, $I_{r}$ from the end effector of the DSRE, the MI value is $MI_{I_{l},I_{r}}=H_{I_{l}}+H_{I_{r}}-H_{I_{l},I_{r}}$ (H is the entropy of image, $H_{I_{l},I_{r}}$ is the joint entropy of images). The MI-based cost function would be computed for each pixel and disparity, resulting in a cost volume that the SGM method would use in subsequent steps to compute the final disparity map.

#### 3.2.2. Aggregate Cost

After the initial cost computation, these costs need to be aggregated across multiple paths through the image to enforce the smoothness constraints while preserving disparity discontinuities, which are often present at object boundaries. A path-wise dynamic programming approach was applied in the SGM algorithm. The cost aggregation step involves accumulating the costs for each pixel along several paths through the image. Typically, 8 paths are considered to cover all directions: left-to-right, right-to-left, top-to-bottom, bottom-to-top, and the four diagonals.

For a given pixel h and disparity d, the aggregated cost $E_{s}(h,d)$ along a path s is calculated as follows:

(5) $$E_{s}(h,d)=C(h,d)+\min\left\{\begin{array}{c} E_{s}(h-s,d)\\ E_{s}(h-s,d-1)+K_{1}\\ E_{s}(h-s,d+1)+K_{1}\\ \underset{i}{\min}E_{s}(h-s,i)+K_{2} \end{array}\right\}-\underset{k}{\min}E_{s}(h-s,k)$$

where C(h,d) is the matching cost at pixel h for disparity d computed from the mutual information, $E_{s}(h-s,d)$ is the cost for pixel h at disparity d accumulated from the previous pixel along path s, $K_{1}$ and $K_{2}$ are the penalty for small disparity changes and for large disparity changes, respectively, to allow for disparity discontinuities. The last term is the minimum cost for the previous pixel h−s over all disparities. After the cost aggregation step, each pixel will have 8 different accumulated costs, 1 for each path. These costs are then combined to produce a single, aggregated cost for each pixel and disparity:

(6) $$E(h,d)=\sum_{s}E_{s}(h,d)$$

The final disparity for each pixel is chosen as the disparity that minimizes this aggregated cost.

#### 3.2.3. Disparity Refinement

**Figure 5:**
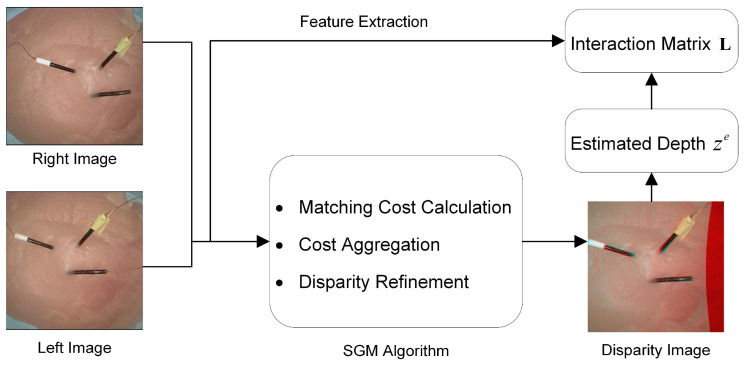
The application of the SGM algorithm on the DSRE system.

Once the initial disparity map is computed, it often requires further refinement to improve the accuracy, especially in occluded or low-texture areas. Refinement steps may include subpixel enhancement, occlusion handling, disparity map filtering, and left–right consistency check. Figure 5 illustrates the application of the SGM algorithm on the DSRE system, which was conducted in the MATLAB R2022a (Mathworks Inc. Portola Valley, CA, USA) with the disparity SGM and reconstruct Scene function. The depth information would be further used in the interaction matrix L in the ASVS controller. This matrix is essential for providing accurate and reliable positional information regarding the target.

### 3.3. Adaptive Stereo Visual Servoing Control

The visual feedback is directly utilized in the control loop of IBVS. The goal is to match the current image with the desired image. The error signal $e\in {\mathbb{R}}^{2}$ for the controller is derived from the difference between the current image features $s\in {\mathbb{R}}^{2}$ and the desired image features $s_{d}$, and $\dot{e}=\dot{s}$.

#### 3.3.1. Monocular Vision Model

Take the left camera as an example to demonstrate the camera model for brevity. The midpoint of the connection line between the two cameras coincides with $O_{t}$.

As shown in Figure 4b, as a given point $Q\in {\mathbb{R}}^{3}$ in $O_{cl}X_{cl}Y_{cl}Z_{cl}$, its coordinates in the image frame $O_{l}x_{l}y_{l}$ and pixel frame $O_{pl}uv$ are $q_{l}={\left(\begin{array}{cc} x_{l} & y_{l} \end{array}\right)}^{T}$ and $s_{l}={\left(\begin{array}{cc} u_{l} & v_{l} \end{array}\right)}^{T}$, respectively. According to the pinhole camera model, the perspective equation can be obtained from the relationship on similar triangles, i.e., as follows:

(7) $$\begin{array}{l} u_{l}=\frac{\lambda_{xl}x_{l}}{\lambda_{l}}+c_{xl}\\ v_{l}=\frac{\lambda_{yl}y_{l}}{\lambda_{l}}+c_{yl} \end{array}$$

where $\lambda_{xl}$, $\lambda_{yl}$ are the focal length in pixels, $c_{xl}$, $c_{yl}$ are the optical center in pixels, and $\lambda_{l}$ is the focal length in millimeters.

The motion of the features ${\dot{s}}_{l}$ on the image plane can be predicted using the interaction matrix and velocity of camera $v_{cl}\in {\mathbb{R}}^{6}$:

(8) $${\dot{s}}_{l}=L_{l}v_{cl}$$

with (7), $L_{l}\in {\mathbb{R}}^{2\times 6}$ is given by

(9) $$L_{l}=\left[\begin{array}{cccccc} -\frac{\lambda_{l}}{z_{cl}^{e}} & 0 & \frac{x_{l}}{z_{cl}^{e}} & \frac{x_{l}y_{l}}{\lambda } & -\frac{\lambda_{l}^{2}+x_{l}^{2}}{\lambda } & y_{l}\\ 0 & -\frac{\lambda_{l}}{z_{cl}^{e}} & \frac{y_{l}}{z_{cl}^{e}} & \frac{\lambda_{l}^{2}+y_{l}^{2}}{\lambda_{l}} & -\frac{x_{l}y_{l}}{\lambda_{l}} & -x_{l} \end{array}\right]$$

where $z_{cl}^{e}$ is the depth of point Q, which can be estimated online with the stereo vision. Similarly, we can obtain the right camera model with ${\dot{s}}_{r}=L_{r}v_{cr}$. 

#### 3.3.2. Stereo Vision Model

Considering the distance *d* between cameras, the transformation matrix Ttcl∈ℝ4×4 from the left camera frame to the robot tip frame is

(10) Ttcl=τx(d/2)=[I3ptcl01],

while the transformation matrix for the right camera is Ttcr=τx(−d/2).

Since the left–right camera is fixed on the tip of the DSRE, the velocity transformation between the camera frame and robot tip frame is given by

(11) vcl=Mtclvtvcr=Mtcrvt

where the transformation matrix Mtc*∈ℝ6×6 can be calculated from [45] with (10), i.e.,

(12) Mtc*=[I3[ptc*]×O3I3]

where [ptc*]× is the skew-symmetric matrix of ptc*.

Let $\dot{s}={\left(\begin{array}{cc} {\dot{s}}_{l} & {\dot{s}}_{r} \end{array}\right)}^{T}$ represent the image features motion extracted from the left and right cameras simultaneously, and the overall velocity transformation from the robot tip frame to the pixel frame can be derived from (8), (11), and (12):

(13) s˙=[LlMtclLrMtcr]vt=Lvt

where $L\in {\mathbb{R}}^{4\times 6}$ is the overall interaction matrix of the stereo vision system.

Inserting (3) in (13), we obtain the relationship of the joint velocity and image feature:

(14) $$\dot{e}=LJ_{r}\dot{\Phi }$$

$LJ_{r}\in {\mathbb{R}}^{4\times 5}$ is a singular matrix without an inverse matrix. Commonly used pseudo-inverse methods often have stability problems. When the robot approaches a singular configuration, this method will cause excessive joint velocities and cause safety problems. The damped least squares method [46] not only improves the image feature tracking effect, but also avoids the singularity problem by limiting joint velocities

(15) $$\min\left({\left\|LJ_{r}\dot{\Phi }-\dot{e}\right\|}^{2}+\sigma {\left\|\dot{\Phi }\right\|}^{2}\right)$$

where σ∈ℝ is a non-zero damping constant.

The inverse problem could be solved by minimizing the object (14). The joint velocities could be derived further as

(16) $$\begin{array}{l} \dot{\Phi }=L_{a}^{†}\dot{e}\\ L_{a}^{†}={\left(LJ_{r}\right)}^{T}{\left(LJ_{r}{\left(LJ_{r}\right)}^{T}+\sigma I\right)}^{-1} \end{array}$$

#### 3.3.3. Adaptive PD Controller

The classic IBVS tasks usually use a proportional control law [28]. Let the error of the feature satisfy the first-order equation $\dot{e}=-\eta e$, so that the error value can decrease rapidly in an exponential speed. Thus,

(17) $$\dot{\Phi }=-\eta L_{a}^{†}e$$

Although a proportional controller responds quickly and is simple to deploy, it cannot eliminate the steady-state error (SSE). In this paper, we adopt a PD controller to reduce the SSE and increase the system stability. Adding a derivative term to (17), we obtain

(18) $$\dot{\Phi }=-\eta L_{a}^{†}e-K_{d}\dot{e}$$

where $K_{d}$ is the non-negative derivative gain.

**Figure 6:**
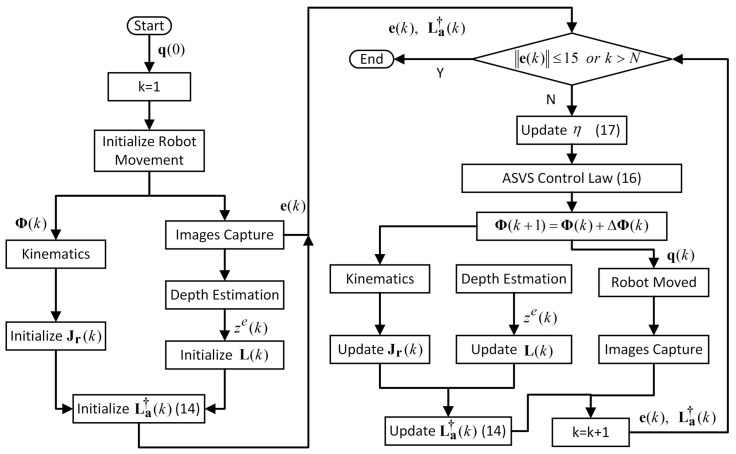
Flow chart of the ASVS controller.

To enhance the efficiency of the IBVS convergence process, we propose the implementation of an adaptive proportional gain. η is dynamically adjusted in real time based on the infinity norm of the feature error, aiming to reduce the number of iterations necessary for convergence and enhance the system’s performance. η is set as follows:

(19) $$\eta =\left(\eta_{u}-\eta_{l}\right)\frac{{\left\|e\right\|}_{\infty }}{{\left\|e_{0}\right\|}_{\infty }}+\eta_{l}$$

where $\eta_{u}$ and $\eta_{l}$ are the preset maximum and minimum gains, respectively, $e_{0}$ is the initial error to a new target. The detailed flow chart of the ASVS controller is shown in Figure 6.

## 4. Experimental Validation

### 4.1. Depth Estimation Validation

**Figure 7:**
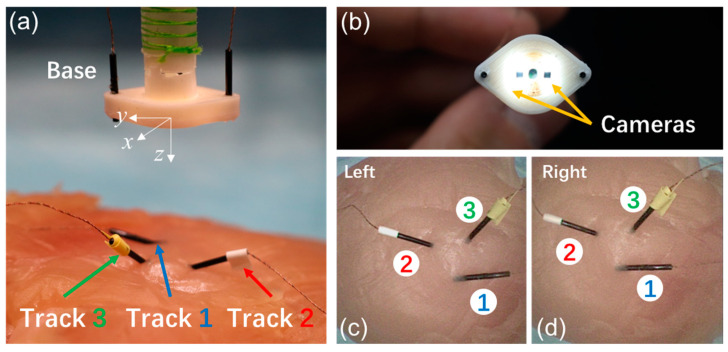
Depth estimation validation setup. (**a**) A base tracker was used to measure the ground truth of depth. (**b**) The stereo vision on the tip of DSRE. (**c**) Image of left camera. (**d**) Image of right camera.

**Figure 8:**
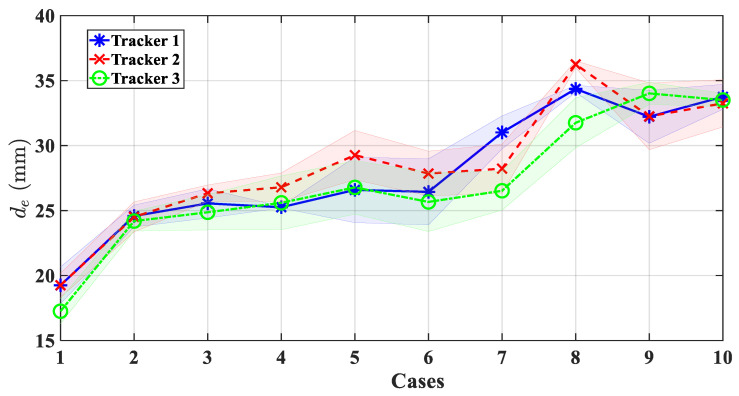
Comparative error band analysis of the camera depth estimation results.

To validate the efficacy of the proposed camera depth estimation algorithm, a comparison was made between the ground truth, derived from EM trackers, model 10001742 (NDI Aurora, Waterloo, ON, Canada), and the values estimated by the algorithm. The specific setup used for the testing process is illustrated in Figure 7a. Three trackers were fixed on a piece of tissue while two base trackers were mounted on the tip of the distal segment. For this experiment, the ground truth was defined as the distance between the base tracker and the trackers placed within the task space. The stereo camera in Figure 7b was first well calibrated with the method in [47], resulting in a notably low mean reprojection error of merely 0.2 pixels. A comparison was then made between the estimated depth, as calculated by the SGM, and the actual values across different altitudes. As depicted in the error band curves in Figure 8, the RMSE of the estimated depth parameters of different markers were 1.5334 mm, 1.5449 mm, and 1.5468 mm, respectively, when compared with the ground truth. This illustrates that the proposed algorithm is highly accurate, and it could obtain reliable depth estimations in various surgical applications. The estimated depth was further used in the visual servoing control.

### 4.2. Controller Performance

The performance of the ASVS was evaluated through the execution of four distinct manipulation tasks in this section. The visual servo controller’s parameters during these tests were established as follows: $q_{0}={\left(\begin{array}{ccccccc} 0 & 0.2 & 0.2 & 0.2 & 0.2 & 0.2 & 0.2 \end{array}\right)}^{T}$, $\eta_{u}=1$, $\eta_{l}=0.5$, σ=0.5, $K_{d}=\mathrm{diag}{\left\{0.02,0.06,0.02,0.06\right\}}^{T}$. In this study, the tracking error was quantified by the Euclidean distance between the current coordinates and target coordinates of the extracted image feature in the left and right frames. The control goal was to stabilize the error below 15 pixels. The frame rate of the left and right cameras was 30 FPS to ensure that the calculated air pressures have been applied to the segments to generate the desired robot shape.

**Figure 9:**
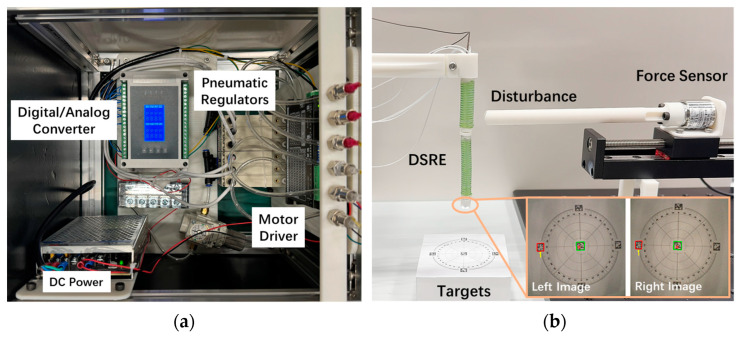
(**a**) The actuator configuration. (**b**) Experimental setup for the ASVS controller performance verification.

As indicated in Figure 9, to simplify the feature detection and extraction process, the stereo vision system was programmed to track the AprilTags [48] in the workspace. Tags with ID 1, 2, 8, and 9 were selected as the tracking targets. The soft robot was controlled to track the target tags to the desired position on the image plane under the air pressure from the pneumatic regulators. A straight beam equipped with a force sensor was fixed on a linear rail guide slide actuator, and the tip caused contact disturbance to the robot. More results can be found in the Supplementary Video S1.

#### 4.2.1. Static Target Tracking

**Figure 10:**
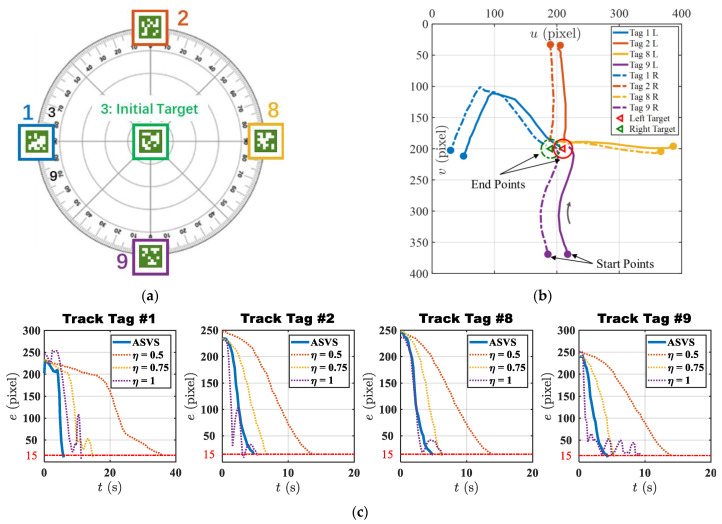
Static target tracking results. (**a**) Distribution configuration of target markers. (**b**) Target movement trajectories in the left and right images, the tags moved from the start points and converged to the end points. (**c**) Tracking errors of different controllers.

In this experiment, the regions of interest (ROIs) within the left and right images were delineated as circular areas centered at points $s_{dl}={\left(\begin{array}{cc} 210 & 200 \end{array}\right)}^{T}$ and $s_{dr}={\left(\begin{array}{cc} 190 & 200 \end{array}\right)}^{T}$ with a radius of 15 pixels, as shown by the red circle and the green dashed circle in Figure 10b. The initial orientation of the DSRE robot was positioned to face the initial target with the AprilTag ID 3. After initialization, the robot was controlled to bring tags with different IDs to the ROI areas. These tags were equidistantly distributed at 90-degree intervals along the circumference of a circle. In each static tag tracking experiment, the adaptively adjusted gains of the ASVS controller were compared with the classic visual servoing method [28] employing fixed gains of η=0.5, η=0.75, and η=1. Figure 10b illustrates the trajectories of various tags in the left and right images, and all ultimately converged within the ROIs. Due to the inherent limitation of robots with inconsistent stiffness in all directions, the DSRE cannot reach the target at some positions, and it will overshoot after passing through these positions, which resulted in the controller’s tracking effect on tag 1 being inferior to several others, as depicted by the blue curve in Figure 10b. Figure 10c and Table 3 detail the pixel errors and convergence times for tracking different tags, respectively. As indicated by (17), when the gain was low, the robot’s end effector moved at a reduced velocity, resulting in excessive time consumption. Conversely, with high gain, the end effector’s velocity increased, potentially leading to overshoot and oscillation. With the gain set at constant values of 5, 0.75, and 1, the classic controller required an average of 19.4 s, 8.29 s, and 9.04 s, respectively, to achieve the tracking of a static target, reducing the error to below 15 pixels. Meanwhile, the proposed control algorithm necessitated only 4.99 s, corresponding to a reduction in time consumption of 74.28%, 39.81%, and 37.94%, respectively. This result illustrates the robustness and reliability of this method in achieving the accurate control of the soft robot arm and precise tracking of the moving target.

#### 4.2.2. Dynamic Target Tracking

**Figure 11:**
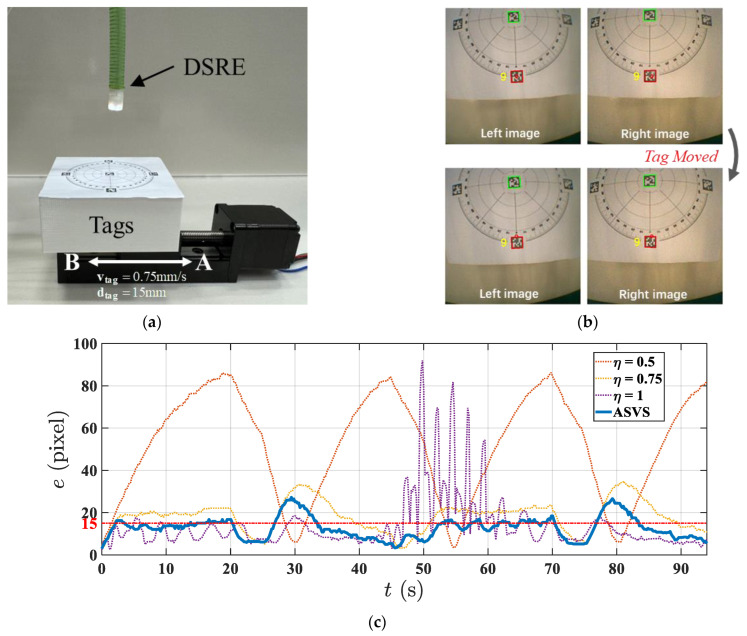
Dynamic target tracking results. (**a**) Experiment setup. (**b**) Endoscopic view when the tag moved. (**c**) Tracking error of different controllers.

This part introduces a tracking experiment designed to evaluate the control performance of the robot under dynamic target changes. As demonstrated in the previous experiment, comparisons were made between the ASVS and classical algorithms using different fixed gains. Figure 11a shows the experimental setup, with tags affixed to an electric linear slide rail, which moved back and forth between two points, A and B, 15 mm apart, at a specified velocity of $v_{tag}=0.75\mathrm{mm}/s$. The endoscopic images captured during the experiment and the corresponding tracking errors are presented in Figure 11b,c, respectively. The robot was tasked to follow tag 9, while the linear guide remained stationary until the initial stable tracking of the target was accomplished by the robot. As shown in Figure 11b, a significant surge in error occurred when the tag’s movement direction changed, but the controller quickly re-captured the tag and subsequently reduced the tracking difference to less than 15 pixels. Table 4 enumerates the tracking errors’ root mean square error (RMSE), mean absolute value error (MAE), standard deviation (SD), and maximum error $e_{\max}$ for different controllers. The ASVS outperformed the classic controller across various metrics, maintaining the RMSE and MAE beneath 15 pixels, with an SD of merely 5.14 pixels. This rapid error correction is indicative of the agility and precision of the real-time tracking of dynamic targets.

#### 4.2.3. Tracking under Unknown External Disturbance

**Figure 12:**
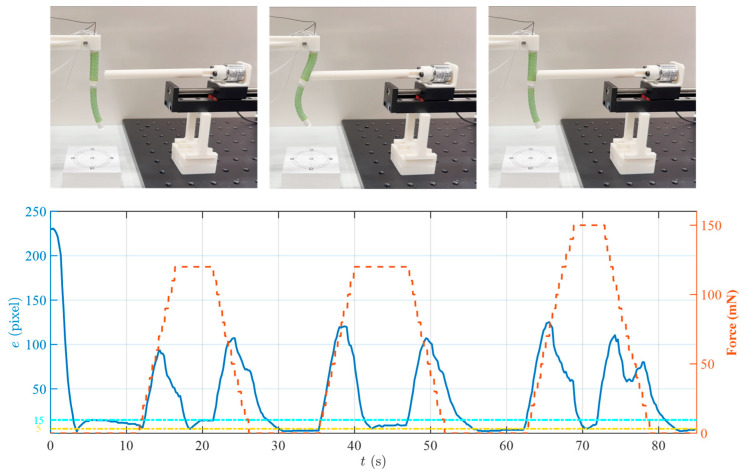
Tracking results under unknown external disturbance. The upper pictures show the robot configurations before, during, and after the external force application. The figure below shows the changes in external force and tracking error results.

The performance of dealing with external disturbance was also evaluated. During the test, the robot utilized endoscopic image feedback exclusively. An external force of unknown magnitude and location was applied to the soft robot using a slender beam affixed with a single-axial force sensor, which caused a mutable robot configuration. The control objective was to bring the target tag into the predefined ROIs on the image planes. The robot configuration and experimental results are shown in Figure 12. During the experiment, at 11.6 s, the robot first received an external force of 120 mN, and the tracking error increased from 5.9 pixels to 93.4 pixels. The controller subsequently engaged to resist the external force and tracked the tag to the ROIs within approximately 3.5 s, reducing the error to less than 15 pixels. After the external force was maintained for 5 s, it was removed, and the controller repeated the above error correction process. During the entire test process, the robot was affected by dynamic external forces a total of three times. The maximum external force was 150 mN. The robot could maintain the tracking of the target and the error could converge to five pixels. This shows that image-based feedback control is efficacious in enabling the DSRE robot to adjust its configuration responsively to external environmental conditions, thereby enhancing the tracking precision.

#### 4.2.4. Trajectory Tracking

**Figure 13:**
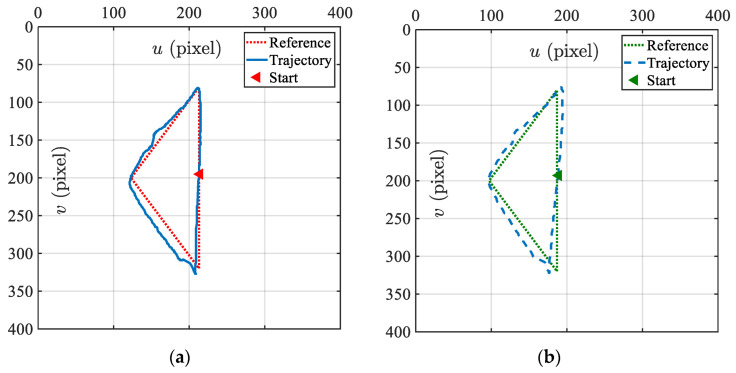
Triangular trajectory tracking results. (**a**) Target movment in the left image. (**b**) Target movment in the right image.

Marking the lesion is an important early step in ESD surgery [7]. The end effector of the soft robot carrying the electrosurgical knife should always be perpendicular to the working surface, marking along the periphery of the target lesion by coagulation. This experiment aims to evaluate the feasibility of robots in the marking process, with the DSRE tracking a triangular reference trajectory. This trajectory guided the tag moving along the pre-defined path in the captured images. The experimental trajectories of the pixel positions on the left and right image planes are shown in Figure 13. The overall RMSE of the left and right image errors was 13.51 pixels, which demonstrates that the controller has a good tracking performance for the trajectories.

### 4.3. Resection in an Ex Vivo Porcine Stomach

**Figure 14:**
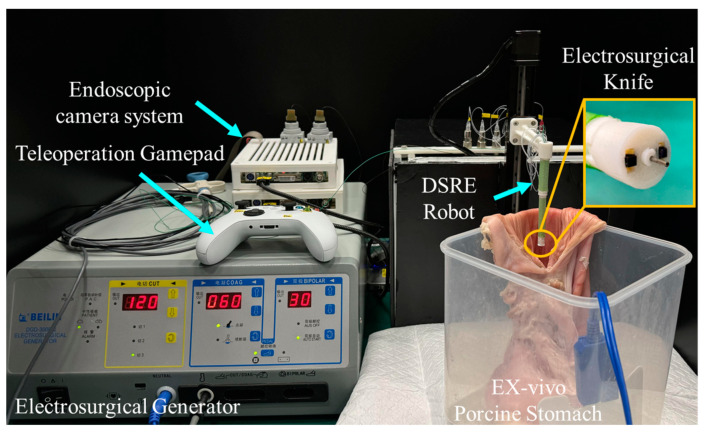
Experimental setup of ex vivo trial in a porcine stomach.

**Figure 15:**
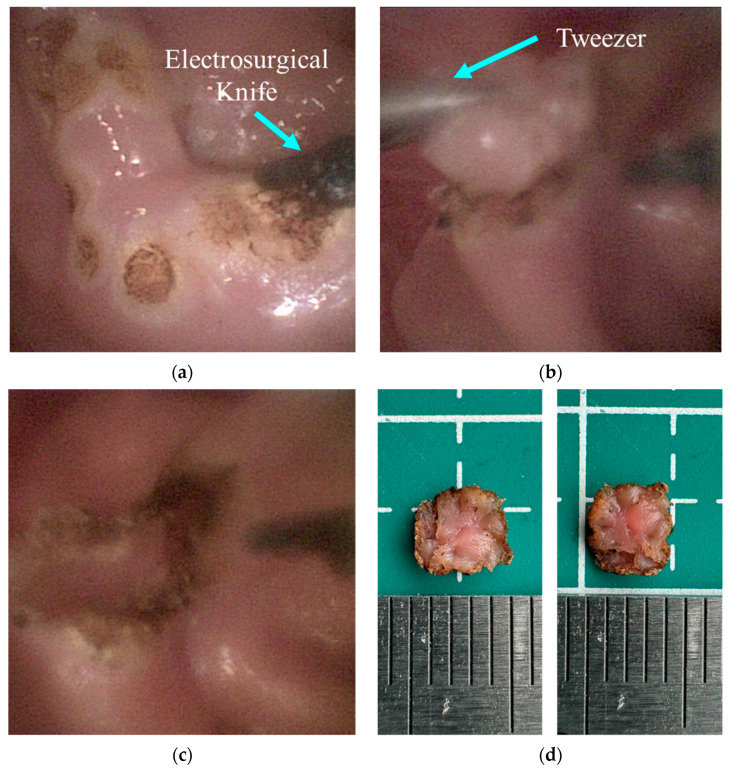
ESD test results in ex vivo porcine stomach. (**a**) Marking points. (**b**) Cutting with assistance. (**c**) Cutting results. (**d**) Target tissue after dissection.

This test was designed to assess the practical efficacy of robotic systems in ESD procedures. As shown in Figure 14, an ex vivo porcine stomach was affixed within a receptacle to simulate surgical conditions. The operator used a gamepad to perform a remote operation program on the DSRE and controlled the robot to complete the ESD surgery in the stomach. Figure 15 shows the entire process of the ESD surgery. According to the DSRE configuration shown in Figure 2c, the angle between the end effector and the horizontal plane was only 12.5°, which illustrates that the robot can cut tissue in multiple directions. Such a small operating angle can improve the cutting efficiency and safety of traditional ESD. Figure 15a,b show the left endoscopic images during ESD marking and cutting, respectively. The operator operated the DSRE to perform electrocoagulation around the lesion and successfully completed a series of marking points. During the cutting process, the operator used tweezers to assist in pulling the target tissue up to expose the cutting point to the robot and used the joystick of the gamepad to adjust the cutting angle of the robot. After the procedure, as illustrated in Figure 15c, the DSRE has completely removed the target tissue mass without causing perforation in the porcine stomach wall. Finally, a piece of tissue measuring approximately 5×4 mm was successfully removed. Therefore, this ex vivo experiment illustrates the feasibility of applying the DSRE in ESD surgery.

## 5. Conclusions

This work presents a novel dual-segment soft robotic endoscope with stereo vision designed to enhance reachability and dexterity during ESD surgery. The proposed system can execute multi-angle cutting operations at a small angle relative to the lesion surface, allowing for efficient en bloc resection. Additionally, the system incorporates two calibrated RGB cameras and a depth estimation algorithm to provide real-time 3D information of the tumor, which is also used to guide the control framework. Based on the adaptively updated gain and PD control laws, the stereo vision servo controller improves the convergence speed and path tracking performance during surgery. The experimental results indicate that the proposed system improves the motion stability and precision. Ex vivo testing further demonstrates its significant potential in endoscopic surgery.

## Figures and Tables

**Table 1:** Comparison of the performance of the DSRE with related robot system.

Study	Backbone	Actuator	Model	Controller	Time	Error
Zhao et al. [22]	Continuous	Pneumatic	PCC	Jacobian estimation	0.18 s	0.62 mm
Zhang et al. [31]	Rigid	-	Geometric	VS with optimization	-	15.4629 Pixel
Roshanfar et al. [32]	Continuous	Pneumatic	Cosserat	-	-	MAE 5.79%
Chen et al. [21]	Continuous	Pneumatic	PCC	Classic IBVS	13 iterations	RMSE 1.199 mm
Lau et al. [33]	Discrete	Cable	PCC	Feedforward	0.25 s	4°
Li et al. [34]	Continuous	Cable	PCC	MPC	-	-
Abdulhafiz et al. [35]	Discrete	Cable	CNN	CNN-based VS	85 iterations	SAD 0.058 mm
Greer et al. [36]	Continuous	sPAMs	PCC	Classic IBVS	-	<5% in 2 s
DSRE in this study	Continuous	Pneumatic	PCC	ASVS	0.19 s	13.51 Pixel

**Table 2:** Comparison of the DSRE system with previous design [21] and conventional endoscopes.

Systems	Actuation Type	Segments	Body Length (mm)	Diameter (mm) ^1^	Max. Angles (°)	Camera	Working Channel (mm)
DSRE	Robotized pneumatic	2	72	P 9, D 7	P170 + D88	2	2
Chen et al. [21]	Robotized pneumatic	2	85	P 10, D 8	P100 + D88	2	s2
CF-XZ1200L/I [39]	Manual cable-driven	1	-	13.2	180	1	3.7
EC-760S-V/L [40]	Manual cable-driven	1	-	12.8	180	1	3.8
EXALT Model D [41]	Manual cable-driven	1	-	15.1	120	1	4.2

^1^ P and D denote proximal and distal, respectively.

**Table 3:** Convergence times of ASVS and the classic control law with different constant η.

Controllers	Tag #1 (s)	Tag #2 (s)	Tag #8 (s)	Tag #9 (s)	Avg. (s)	Dec. (%)
ASVS	5.89	4.78	4.93	4.36	4.99	-
η=0.5	35.87	13.80	13.87	14.05	19.40	74.28
η=0.75	14.78	6.86	6.22	5.31	8.29	39.81
η=1	11.21	5.33	6.29	9.33	8.04	37.94

**Table 4:** Control performance of ASVS and the classic control law with different constant η in [28].

Controller	RMSE		MAE		SD		$e_{\max}$	
	Data (pixel)	Dec. (%)	Data (pixel)	Dec. (%)	Data (pixel)	Dec. (%)	Data (pixel)	Dec. (%)
ASVS	13.48	-	12.46	-	5.14	-	27.24	-
η=0.5	57.63	76.57	52.06	76.07	24.74	79.22	86.20	68.40
η=0.75	19.37	30.41	17.88	30.31	7.44	30.91	34.91	21.97
η=1	18.65	27.72	13.88	10.23	12.47	58.78	91.95	70.38

## Data Availability

Data are contained within the article.

## Supplementary Materials

The following supporting information can be downloaded at: https://www.mdpi.com/article/10.3390/mi15020276/s1, Video S1: the experimental results of the dual-segment robotic endoscope (DSRE).

## Appendix A

The translation and rotation matrices in (2) are given by

(A1) $$\begin{array}{c} R_{y}(\theta )=\left[\begin{array}{cccc} c\theta & 0 & s\theta & 0\\ 0 & 1 & 0 & 0\\ -s\theta & 0 & c\theta & 0\\ 0 & 0 & 0 & 1 \end{array}\right],R_{z}(\varphi )=\left[\begin{array}{cccc} c\varphi & -s\varphi & 0 & 0\\ s\varphi & c\varphi & 0 & 0\\ 0 & 0 & 1 & 0\\ 0 & 0 & 0 & 1 \end{array}\right],\\ \tau_{x}(t)=\left[\begin{array}{cccc} 1 & 0 & 0 & t\\ 0 & 1 & 0 & 0\\ 0 & 0 & 1 & 0\\ 0 & 0 & 0 & 1 \end{array}\right],\tau_{z}(t)=\left[\begin{array}{cccc} 1 & 0 & 0 & 0\\ 0 & 1 & 0 & 0\\ 0 & 0 & 1 & t\\ 0 & 0 & 0 & 1 \end{array}\right] \end{array}$$

In order to simplify the formula, use *s* and *c* instead of sine and cosine functions, and the forward kinematics in (2) can be extended to

(A2) Ttb=[RtbPtb01]=[T11T12T13T14T21T22T23T24T31T32T33T340001]=[−c(θe)σ4−σ1s(φe)σ8−c(φe)σ9σ2−s(θe)σ4le(c(θe)σ4+σ1)θe−td(s(θe)σ4−σ2)+lsc(φs)θs+zec(φs)s(θs)−leσ4θe−lsc(θs)c(φs)θsσ5c(φe)σ12−s(φe)σ13σ6tdσ6−leσ5θe+lss(φs)θs+zes(θs)s(φs)+leσ10θe−lsc(θs)s(φs)θs−σ3−c(θe)σ11c(φe)s(θs)σ14+s(θs)σ15s(φe)σ7zs+tdσ7+zec(θs)−leσ11θe+lss(θs)θs+le(σ3+c(θe)σ11)θe0001]

where

$$\begin{array}{l} \sigma_{1}=c\left(\varphi_{s}\right)s\left(\theta_{e}\right)s\left(\theta_{s}\right),\;\sigma_{2}=c\left(\theta_{e}\right)c\left(\varphi_{s}\right)s\left(\theta_{s}\right),\;\sigma_{3}=c\left(\theta_{s}\right)s\left(\theta_{e}\right),\;\\ \sigma_{4}=c\left(\varphi_{e}\right)\sigma_{8}+s\left(\varphi_{e}\right)\sigma_{9},\;\sigma_{5}=c\left(\theta_{e}\right)\sigma_{10}-s\left(\theta_{e}\right)s\left(\theta_{s}\right)s\left(\varphi_{s}\right),\;\\ \sigma_{6}=s\left(\theta_{e}\right)\sigma_{10}+c\left(\theta_{e}\right)s\left(\theta_{s}\right)s\left(\varphi_{s}\right),\;\sigma_{7}=c\left(\theta_{e}\right)c\left(\theta_{s}\right)-s\left(\theta_{e}\right)\sigma_{11}\\ \sigma_{8}=s\left(\varphi_{s}\right)\sigma_{14}-c\left(\theta_{s}\right)c\left(\varphi_{s}\right)\sigma_{15},\;\sigma_{9}=\sigma_{15}s\left(\varphi_{s}\right)+c\left(\theta_{s}\right)c\left(\varphi_{s}\right)\sigma_{14},\;\\ \sigma_{10}=c\left(\varphi_{e}\right)\sigma_{13}+s\left(\varphi_{e}\right)\sigma_{12},\;\sigma_{11}=c\left(\varphi_{e}\right)s\left(\theta_{s}\right)\sigma_{15}-s\left(\theta_{s}\right)s\left(\varphi_{e}\right)\sigma_{14},\;\\ \sigma_{12}=c\left(\varphi_{s}\right)\sigma_{15}-c\left(\theta_{s}\right)s\left(\varphi_{s}\right)\sigma_{14},\;\sigma_{13}=c\left(\varphi_{s}\right)\sigma_{14}+c\left(\theta_{s}\right)\sigma_{15}s\left(\varphi_{s}\right),\;\sigma_{14}=s\left(\varphi_{e}+\frac{\pi }{3}\right). \end{array}$$

The pose $\Theta_{t}\in {\mathbb{R}}^{6}$ of the robot end effector can be calculated from (A2)

(A3) Θt=[PtbΩtb]T=[PxPyPzΩxΩyΩz]TPx=T14, Py=T24, Pz=T34Ωx=atan2(T32, T33), Ωy=atan2(−T31, T322+T332), Ωz=atan2(T21, T11)

The velocity $v_{t}\in {\mathbb{R}}^{6}$ of the robot end effector is

(A4) $$v_{t}={\dot{\Theta }}_{t}={\left[\begin{array}{cccccc} \tau_{x} & \tau_{y} & \tau_{z} & \omega_{x} & \omega_{y} & \omega_{z} \end{array}\right]}^{T}$$

Thus, the Jacobian matrix $J_{r}\in {\mathbb{R}}^{6\times 5}$ in (3) is used to represent the approximate linear relationship between the robot tip velocity $v_{t}$ and joint velocity $\dot{\Phi }$, which can be derived from the forward kinematics as follows:

(A5) $$\begin{array}{l} v_{t}=J_{r}\dot{\Phi }\\ \left[\begin{array}{c} \tau_{x}\\ \tau_{y}\\ \tau_{z}\\ \omega_{x}\\ \omega_{y}\\ \omega_{z} \end{array}\right]=\left[\begin{array}{ccccc} \frac{\partial \tau_{x}}{\partial z_{s}} & \frac{\partial \tau_{x}}{\partial \theta_{s}} & \frac{\partial \tau_{x}}{\partial \varphi_{s}} & \frac{\partial \tau_{x}}{\partial \theta_{e}} & \frac{\partial \tau_{x}}{\partial \varphi_{e}}\\ \frac{\partial \tau_{y}}{\partial z_{s}} & \frac{\partial \tau_{y}}{\partial \theta_{s}} & \frac{\partial \tau_{y}}{\partial \varphi_{s}} & \frac{\partial \tau_{y}}{\partial \theta_{e}} & \frac{\partial \tau_{y}}{\partial \varphi_{e}}\\ \ldots & \ldots & \ldots & \ldots & \ldots \\ \frac{\partial \omega_{z}}{\partial z_{s}} & \frac{\partial \omega_{z}}{\partial \theta_{s}} & \frac{\partial \omega_{z}}{\partial \varphi_{s}} & \frac{\partial \omega_{z}}{\partial \theta_{e}} & \frac{\partial \omega_{z}}{\partial \varphi_{e}} \end{array}\right]{\left[\begin{array}{r} z_{s}\\ \theta_{s}\\ \varphi_{s}\\ \theta_{e}\\ \varphi_{e} \end{array}\right]}^{\prime } \end{array}$$
